# Validation of an instrument to assess knowledge, attitudes, and practices regarding occupational accident prevention among rice farmers

**DOI:** 10.1371/journal.pone.0337550

**Published:** 2025-11-26

**Authors:** My Ha Nguyen, Toan Van Ngo, Linh Gia Vu, Dat Cong Truong, Hai Minh Vu

**Affiliations:** 1 Department of Health Organization and Management, Faculty of Public Health, Thai Binh University of Medicine and Pharmacy, Hung Yen, Vietnam; 2 Institute for Preventive Medicine and Public Health, Hanoi Medical University, Hanoi, Vietnam; 3 Health Strategy and Policy Institute, Hanoi, Vietnam; 4 Department of Environmental Health, Faculty of Public Health, Thai Binh University of Medicine and Pharmacy, Hung Yen, Vietnam; 5 Department of Trauma, Thai Binh University of Medicine and Pharmacy, Hung Yen, Vietnam; Canadian University Dubai, UNITED ARAB EMIRATES

## Abstract

**Background:**

Despite the hazardous nature of rice farming, limited evidence exists regarding farmers’ knowledge, attitudes, and practices (KAP) toward accident prevention, and no standardized instrument is currently available to measure these dimensions in Vietnam or comparable contexts. This study aimed to develop and validate a psychometrically sound instrument for assessing rice farmers’ KAP related to occupational accident prevention.

**Methods:**

Instrument development and validation were carried out in three stages. First, an initial item pool was generated through an extensive review of existing literature. Second, content validity was established through expert consultation involving professionals in agriculture, occupational health, and public health. Third, a pilot study was conducted with 168 rice farmers in Thai Binh Province, Vietnam, to evaluate the instrument’s psychometric properties.

**Results:**

Exploratory factor analysis identified 20 items across five factors in the knowledge domain, nine items forming a single factor for attitudes, and 17 items grouped into four factors for practices, explaining 85.8%, 43.8%, and 72.3% of the total variance, respectively. Confirmatory factor analysis supported these structures, demonstrating satisfactory model fit across domains. The instrument exhibited high internal consistency, with Cronbach’s alpha values ranging from 0.894 to 0.969 for knowledge, 0.833 for attitudes, and 0.805 to 0.933 for practices.

**Conclusion:**

The validated instrument provides a reliable and valid measure of rice farmers’ knowledge, attitudes, and practices concerning occupational accident prevention. It offers a robust foundation for future research, monitoring, and targeted interventions aimed at improving safety behaviors and reducing injury risks among agricultural workers.

## Introduction

The increasing incidence of occupational injuries in agricultural production has become a major public health concern, reflecting persistent challenges in ensuring farmers’ safety and well-being. Behavioral factors play a decisive role in the occurrence of occupational accidents and injuries; consequently, farmers’ knowledge, attitudes, and practices (KAP) regarding occupational accident prevention are critical determinants of work-related risk reduction [1]. Inadequate awareness and insufficient education on occupational safety have been consistently identified as important contributors to injury occurrence in agricultural settings [2]. Prior research has conceptualized risk perception as an individual’s subjective evaluation of the potential consequences of an accident or hazardous event [3]. Farmers who possess heightened risk perception are more capable of recognizing the nature and severity of occupational hazards, thereby enabling more informed and preventive behavioral responses. Typically, farmers manage injury-related risks through adaptive decision-making shaped by previous experiences with accidents and injuries [4].

The interrelationship among knowledge, attitudes, and practices is well established and mutually reinforcing [5]. Deficient knowledge of safe work procedures represents a major causal factor in occupational accidents, while limited awareness of workplace hazards can precipitate farm-related incidents [6]. Empirical evidence indicates that farmers who have not received systematic training in the appropriate use of personal protective equipment (PPE) exhibit higher rates of occupational accidents, injuries, and work-related illnesses [7]. Accordingly, individuals engaged in agricultural production must undergo structured training and continuous supervision in occupational safety. Competent personnel should undertake comprehensive risk assessments of work tasks and environmental conditions, and use the results to identify appropriate preventive and control measures. Regular occupational safety and health training enhances farmers’ knowledge, skills, and attitudes toward safety, which, in turn, has been shown to reduce the frequency and severity of occupational injuries in agricultural work [8–12].

KAP assessments have long been utilized as standardized approaches to examine knowledge, attitudes, and behavioral practices within specific populations across diverse disciplines, including healthcare, education, social sciences, and occupational safety and health [12–17]. Because KAP-based questionnaires can be adapted to context-specific conditions, they provide valuable insights into workers’ safety-related competencies within the rice-farming sector [18,19]. Knowledge assessments facilitate the identification of deficiencies in understanding, thereby supporting the design of targeted educational interventions to improve awareness of occupational accident prevention [20]. Evaluating attitudes is essential for fostering a positive safety culture, encouraging compliance with safety regulations, and promoting proactive hazard reporting and safe work practices [21,22]. Examination of practical behaviors further reveals inconsistencies between knowledge and practice, creating opportunities for corrective actions that align daily work behavior with established preventive measures [10,23,24].

Despite the recognized importance of KAP in understanding safety behaviors, there remains a lack of standardized, psychometrically validated tools for assessing farmers’ KAP regarding occupational accident prevention in rice farming. Existing questionnaires do not adequately capture the multidimensional and context-specific aspects of safety within this occupational group. Therefore, the development of a reliable and valid measurement instrument tailored to rice farmers is essential for accurately evaluating their KAP and informing evidence-based interventions. The present study aimed to develop and validate a KAP instrument for Vietnamese rice farmers in two stages: Stage I involved the development of the initial instrument, including item generation and expert review; Stage II comprised a pilot study and psychometric validation using Exploratory Factor Analysis (EFA) and Confirmatory Factor Analysis (CFA).

## Materials and methods

### Study design and participants

A pilot study was conducted from January to April 2024 in several villages located in Dong Hung District, Thai Binh Province, Vietnam. The study population comprised rice farmers who met the following inclusion criteria: (1) aged 18 years or older; (2) directly engaged in rice farming activities within the study area for at least 12 months; and (3) fulfilling the agricultural labor standards outlined in the International Labour Organization (ILO) Convention No. 184 on Safety and Health in Agriculture [25].

The initial version of the instrument contained 50 items. The required sample size for the pilot study was determined according to the recommendations of Mvududu and Sink (2013), who proposed a participant-to-item ratio ranging from 3:1–20:1 for EFA [26,27]. In addition, Field et al. (2018) emphasized that the total sample should ideally exceed 100 participants to ensure stable factor extraction and adequate statistical power [28]. Taking into account these guidelines and available logistical resources, a ratio of three participants per item was adopted, yielding a minimum required sample size of 150 respondents. Ultimately, 168 rice farmers were successfully recruited and completed the survey during the study period.

### Data measurement

The development and validation of the instrument to assess rice farmers’ KAP regarding occupational accident prevention were conducted in three sequential steps. The research was based on the following steps proposed by DeVellis [29]:

### Step 1: Development of the initial instrument

The initial pool of items was constructed based on a comprehensive review of the literature and international guidelines on agricultural occupational safety and health. Specifically, key references and frameworks were drawn from previous studies [20,30–36], the International Labour Organization (ILO) Convention No. 184 on Safety and Health in Agriculture [37] and relevant World Health Organization (WHO) documents addressing occupational accident prevention in farming contexts [38]. Additional insights were obtained from prior KAP-based studies in agriculture and occupational safety, which informed the operationalization of the three core domains—knowledge, attitudes, and practices—and their corresponding subdomains (e.g., physical hazards, chemical exposure, ergonomics, and animal-related risks).

No existing validated KAP instrument specific to rice farmers or agricultural accident prevention in Vietnam was identified. Therefore, the initial 50-item pool was developed by synthesizing concepts and items from multiple international and national documents to ensure comprehensive domain coverage and contextual relevance. The preliminary version of the questionnaire included 21 knowledge items, 9 attitude items, and 20 practice items.

### Step 2: Face validity and expert evaluation

Before conducting content validation, face validity was performed with a small group of 10 rice farmers from the target community to evaluate the clarity, comprehensibility, and cultural appropriateness of all items. Based on participants’ feedback, one item was removed due to cultural unsuitability, resulting in a 49-item instrument.

Following the face validation, content validity was assessed by a multidisciplinary panel of seven experts, including specialists in agriculture (n = 2), occupational health and safety (n = 2), trauma and injury prevention (n = 1), and public health (n = 2). Each expert independently rated the relevance, clarity, and representativeness of every item using a four-point Likert scale (1 = not relevant to 4 = highly relevant).

A Content Validity Index (CVI) was then computed at both the item level (I-CVI) and scale level (S-CVI) to quantify the degree of expert agreement. Items with I-CVI values below 0.78 were reviewed and revised for clarity or removed if necessary, while the S-CVI was used to evaluate the overall adequacy of the instrument’s content coverage. Feedback from the expert panel was also qualitatively analyzed to refine item wording, eliminate redundancies, and ensure conceptual alignment with occupational accident prevention principles in rice farming.

### Step 3: Pilot testing and psychometric validation

The refined 49-item instrument was subsequently subjected to pilot testing and psychometric evaluation, including EFA and CFA, as detailed in the Statistical Analysis section.

The pilot survey was conducted in Dong Hung District, Thai Binh Province, Vietnam. Eligible rice farmers were selected through a random lottery process based on predefined inclusion and exclusion criteria. Additional participants were subsequently identified using a neighbor-household approach until the required sample size was achieved. Data collection was carried out through structured, face-to-face interviews conducted by trained field investigators who had received prior instruction on interview techniques and ethical procedures.

During the pilot phase, the pretested questionnaire was administered to assess the clarity, comprehension, and relevance of each item within the target population. Feedback from respondents was used to refine the instrument further, particularly in terms of linguistic clarity and cultural appropriateness. The collected data were then subjected to psychometric evaluation, including assessments of construct validity and internal consistency reliability, to ensure that the final instrument met accepted methodological and statistical standards.

### Statistical analysis

Data analysis was performed using Stata version 17.0 and AMOS version 20.0 (IBM Corp., Armonk, NY, USA). The psychometric validation of the instrument was conducted through a series of statistical procedures designed to assess construct validity, model fit, and internal consistency reliability.

EFA was first employed to identify the underlying factor structure and assess construct validity. The factor extraction was conducted using the Principal Axis Factoring (PAF) method with an oblimin rotation, allowing for correlation among factors. Items were retained if they met the following criteria: factor loadings ≥ 0.45, communality values ≥ 0.30, and cross-loading differences ≥ 0.20 between primary and secondary factors. Items that failed to meet these thresholds were removed to improve conceptual clarity and measurement precision. To support factor retention decisions, both the Kaiser criterion (eigenvalues > 1) and parallel analysis were performed. Prior to conducting EFA, the Kaiser–Meyer–Olkin (KMO) measure of sampling adequacy and Bartlett’s test of sphericity were applied to confirm data suitability for factor analysis. A KMO value above 0.6 and a statistically significant Bartlett’s test (p < 0.05) were considered indicative of adequate inter-item correlations and sampling adequacy [39,40]. Factor loadings, standard errors, and modification indices were examined to evaluate the measurement quality and model specification.

Following EFA, CFA was conducted to verify the factor structure identified in the exploratory stage and to evaluate overall model fit. Given the ordinal nature of the Likert-type items and slight deviations from multivariate normality, data distribution was first examined using Mardia’s coefficient and univariate skewness and kurtosis statistics. The CFA was then performed using the Weighted Least Squares Mean and Variance adjusted (WLSMV) estimation method, which is robust for non-normally distributed ordinal data. The Satorra–Bentler correction was applied to further adjust for non-normality. Model fit was evaluated using multiple indices and their conventional cutoff values [32]: χ²/df ≤ 3.0 for relative chi-square, RMSEA ≤ 0.08 for approximate fit, and CFI ≥ 0.90 for comparative fit.

Internal consistency reliability was assessed using Cronbach’s alpha and McDonald’s omega (ω) coefficients, with values ≥ 0.70 considered acceptable. Item-total correlations and “alpha if item deleted” values were also computed to evaluate the homogeneity of each domain and the contribution of individual items to overall reliability. All analyses were performed separately for the knowledge, attitude, and practice domains to ensure domain-specific reliability and construct validity.

### Ethical considerations

All procedures involving human participants adhered to the ethical standards of the relevant national and institutional research committees and were conducted in accordance with the principles outlined in the Declaration of Helsinki (1975, as revised in 2000). Ethical approval for the study was obtained from the Ethics Committee of Hanoi Medical University (Approval No. 1054/GCN-HMUIRB; Project Code: TNLDLUA). Permission to conduct the study was also granted by the local authorities of the research sites in Dong Hung District, Thai Binh Province. Participation in the study was entirely voluntary. All eligible farmers were informed about the study objectives, procedures, potential risks, and benefits prior to data collection. Written informed consent was obtained from each participant before enrollment. Confidentiality and anonymity were maintained throughout the study by assigning coded identifiers to participants and storing all data securely.

## Results

A total of 168 rice farmers participated in the study, comprising 42.3% males and 57.7% females. The mean age of participants was 59.07 ± 10.86 years, with more than half (51.8%) aged 60 years or older. The average duration of engagement in rice farming was 35.21 ± 13.47 years. On average, participants reported spending approximately 3.99 ± 2.14 hours per day performing rice cultivation activities (Table 1).

**Table 1:** General characteristics of study participants.

Characteristics	Categories	Freq. (n)	Percent (%)
Total		168	100.0
Gender	Male	71	42.3
	Female	97	57.7
Age group (years)	< 40	8	4.8
	40–49	25	14.9
	50–59	48	28.6
	≥ 60	87	51.8
Mean age (Mean ± SD)			59.07 ± 10.86
Duration in rice farming (years)			35.21 ± 13.47
Daily time spent on rice farming (hours)			3.99 ± 2.14

The EFA for the knowledge domain identified five distinct factors representing different aspects of occupational accident prevention among rice farmers: prevention of physical hazards, plant protection chemical (PPC)–related accidents, equipment and machinery safety, ergonomic hazards, and animal-related accidents. The factor loadings ranged from 0.788 to 0.954, indicating strong associations between items and their respective constructs. The Kaiser–Meyer–Olkin (KMO) measure of 0.862 and a significant Bartlett’s test (p < 0.001) confirmed sampling adequacy and data suitability for factor analysis. The five extracted factors accounted for 85.85% of the total variance. Cronbach’s alpha values for each factor ranged from 0.852 to 0.969, while the overall reliability for the knowledge scale was 0.894, with McDonald’s ω = 0.815, demonstrating high internal consistency across all domains (Table 2).

**Table 2:** Results of exploratory factor analysis for the knowledge domain and Cronbach’s alpha values for each factor.

Factor	Items	Factor Loading	Communalities	Item–Total Correlation	Alpha if Item Deleted	Cronbach’s Alpha
F1: Prevention of accidents due to physical hazards	KT12 – Avoid working during peak midday heat (11 a.m.–2 p.m.)	0.951	0.9633	0.6215	0.8720	0.969
	KT8 – Use anti-slip boots and shoes when working in the field	0.940	0.9361	0.6125	0.8722	
	KT11 – Drink sufficient water while working	0.938	0.9398	0.6285	0.8717	
	KT10 – Wear long, breathable clothing while working in the field	0.872	0.8698	0.6548	0.8711	
	KT9 – Wear hats or caps when working outdoors	0.855	0.7669	0.5508	0.8741	
F2: Prevention of accidents due to plant protection chemicals (PPC)	KT18 – Change clothes and shower after spraying or mixing PPC	0.901	0.8263	0.4742	0.8766	0.949
	KT14 – Use pesticides with labels and valid expiration dates	0.897	0.8513	0.5433	0.8748	
	KT16 – Store PPC separately in clearly labeled containers	0.897	0.8511	0.5607	0.8743	
	KT15 – Wear protective gear when handling PPC	0.890	0.8511	0.5216	0.8753	
	KT17 – Avoid talking, eating, or drinking during PPC application	0.876	0.8298	0.5782	0.8738	
F3: Prevention of equipment-, machinery-, and tool-related accidents	KT1 – Read safety instructions before operating machinery	0.921	0.8668	0.4598	0.8772	0.932
	KT3 – Use appropriate protective gear when operating machinery	0.920	0.8658	0.4592	0.8772	
	KT4 – Ensure electrical safety in the work area	0.895	0.8248	0.4746	0.8765	
	KT2 – Maintain and service equipment regularly	0.864	0.7758	0.4222	0.8783	
F4: Prevention of ergonomic hazards	KT20 – Lift and move heavy objects correctly	0.954	0.9694	0.5253	0.8748	0.967
	KT19 – Change working posture frequently	0.950	0.9504	0.5021	0.8756	
	KT21 – Perform stretching or muscle relaxation exercises	0.917	0.8899	0.4930	0.8759	
F5: Prevention of animal-related accidents	KT7 – Avoid striking or pushing animals	0.881	0.8559	0.4561	0.8770	0.852
	KT5 – Stay calm and move carefully around animals	0.860	0.7954	0.3931	0.8787	
	KT6 – Avoid loud noises when handling animals	0.788	0.7092	0.4356	0.8776	

KMO = 0.862; Bartlett’s test of sphericity: p < 0.001

Eigenvalues: 7.024, 3.371, 2.912, 2.234, 1.629

Cumulative variance: 85.85%

Overall α = 0.894

McDonald’s ω = 0.815

The EFA for the attitude domain yielded a single factor comprising nine items that collectively measured farmers’ attitudes toward occupational accident prevention. Factor loadings ranged from 0.516 to 0.794, demonstrating satisfactory convergence of items on the underlying construct. The KMO value of 0.890 and a statistically significant Bartlett’s test (p < 0.001) confirmed the adequacy of the sample for factor analysis. The extracted factor had an eigenvalue of 3.946 and explained 43.85% of the total variance. Reliability analysis showed a Cronbach’s alpha coefficient of 0.833 and McDonald’s ω = 0.839, both indicating good internal consistency. Item–total correlations ranged from 0.4062 to 0.6947, with alpha if item deleted values between 0.7978 and 0.8312, confirming the stability of the scale (Table 3).

**Table 3:** Results of exploratory factor analysis for the attitude domain and Cronbach’s alpha values.

Item Code	Item Description	Factor Loading	Communalities	Item–Total Correlation	Alpha if Item Deleted
TD1	Occupational accidents in rice production are a serious problem	0.794	0.6302	0.6947	0.7978
TD2	Any farmer is at risk of accidents during rice production	0.758	0.5739	0.6409	0.8037
TD7	Training to enhance occupational safety knowledge and practices is essential	0.755	0.5697	0.6468	0.8040
TD3	Personal safety during rice production is of utmost importance	0.671	0.4498	0.5578	0.8135
TD5	Maintaining a safe work environment helps reduce accident risk	0.659	0.4346	0.5369	0.8159
TD6	I am willing to engage in activities to prevent accidents in rice production	0.648	0.4195	0.5364	0.8159
TD4	Occupational accidents in rice production can be prevented	0.564	0.3194	0.4453	0.8257
TD8	Seeking immediate medical attention after an accident is necessary	0.532	0.2830	0.4228	0.8299
TD9	I am willing to remind or advise others to follow safety measures	0.516	0.2661	0.4062	0.8312

Cronbach’s alpha (9 items) = 0.833

KMO = 0.890; Bartlett’s test: p < 0.001;

Eigenvalue = 3.946;

Cumulative variance = 43.85%

McDonald’s ω = 0.839

The EFA for the practice domain extracted four factors that captured key behavioral dimensions related to occupational accident prevention: occupational protective safety, health maintenance during work, safe use of plant protection chemicals, and machinery and equipment safety. Factor loadings ranged from 0.620 to 0.933, indicating strong relationships between items and their respective constructs. The KMO value of 0.830 and a significant Bartlett’s test (p < 0.001) confirmed data adequacy for factor analysis. The four extracted factors explained 72.25% of the total variance. Cronbach’s alpha coefficients ranged from 0.805 to 0.933 across the four domains, with an overall α of 0.842 and McDonald’s ω = 0.730, signifying satisfactory internal consistency. Item–total correlations ranged from 0.2699 to 0.6445, and the “alpha if item deleted” values (0.792–0.814) indicated that all items contributed meaningfully to the reliability of the practice scale (Table 4).

**Table 4:** Results of exploratory factor analysis for the practice domain and Cronbach’s alpha values for each factor.

Factor	Items	Factor Loading	Communalities	Item–Total Correlation	Alpha if Item Deleted	Cronbach’s Alpha
F1: Occupational protective safety	TH13 – Use protective clothing when exposed to plant protection chemicals (PPC)	0.933	0.8864	0.6254	0.7938	0.933
	TH15 – Wear long, breathable clothing and a hat when working outdoors	0.926	0.8638	0.6327	0.7933	
	TH14 – Wear protective boots	0.908	0.8344	0.6445	0.7922	
	TH11 – Use goggles to protect the eyes	0.896	0.8153	0.5636	0.7985	
	TH12 – Wear protective gloves	0.877	0.7914	0.6233	0.7939	
	TH10 – Wear a face mask	0.620	0.4683	0.4438	0.8059	
F2: Health maintenance during work	TH17 – Exercise regularly	0.843	0.7215	0.2914	0.8139	0.819
	TH16 – Undergo routine health examinations	0.835	0.7094	0.2699	0.8146	
	TH18 – Maintain a nutritious diet and adequate hydration; limit stimulants (e.g., alcohol, beer)	0.778	0.6222	0.2653	0.8147	
	TH19 – Clean the body thoroughly after working	0.751	0.5897	0.3351	0.8118	
F3: Safety in using plant protection chemicals (PPC)	TH6 – Use safe PPC with complete labeling and within expiration dates	0.817	0.7182	0.3690	0.8103	0.805
	TH7 – Avoid eating, drinking, or talking while mixing or spraying PPC	0.792	0.6422	0.3936	0.8092	
	TH8 – Store PPC in a designated, separate area	0.744	0.6557	0.3970	0.8092	
	TH9 – Maintain complete records of PPC usage	0.725	0.5214	0.3824	0.8094	
F4: Machinery and equipment safety	TH4 – Ensure power sources connected to equipment are properly shielded	0.860	0.7206	0.3319	0.8122	0.864
	TH2 – Regularly maintain and service machinery and equipment	0.858	0.7546	0.3231	0.8124	
	TH1 – Read operating instructions carefully before use	0.853	0.8025	0.4218	0.8072	

KMO = 0.830; Bartlett’s test of sphericity: p < 0.001;

Eigenvalues = F1: 4.964, F2: 3.490, F3: 2.448, F4: 1.380

Cumulative variance = 72.25%

Overall Cronbach’s α = 0.842

McDonald’s ω = 0.730

**Fig 1:**
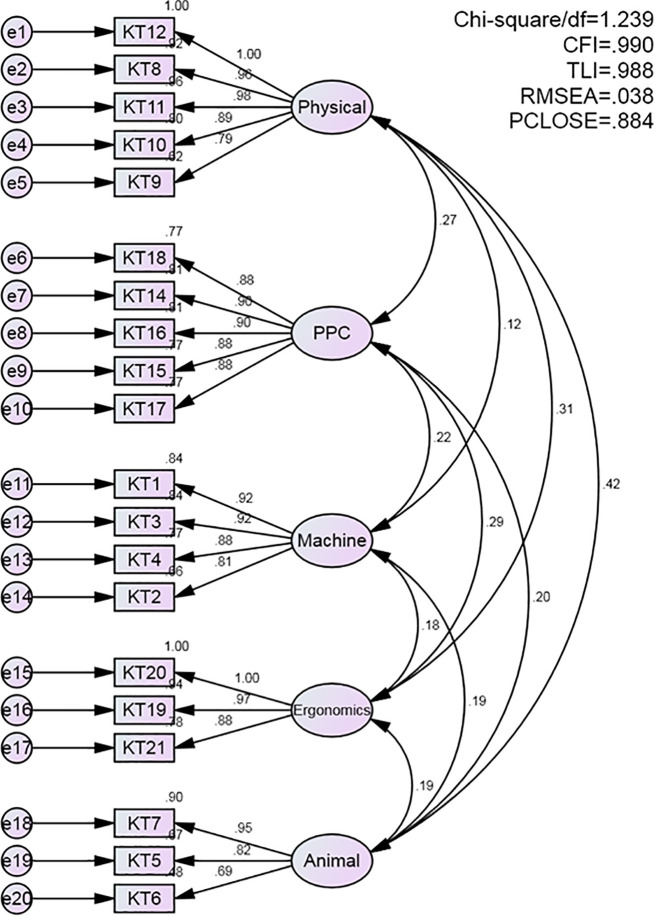
Confirmatory factor analysis model for the knowledge domain. For the attitude domain, the model exhibited an excellent fit, with CMIN/df = 1.030, CFI = 0.998, RMSEA = 0.013, and PCLOSE = 0.859 (Fig 2).

The CFA results supported the construct validity of the instrument across all three domains. For the knowledge domain, the model demonstrated a good fit, with CMIN/df = 1.239, CFI = 0.990, RMSEA = 0.038, and PCLOSE = 0.884 (Fig 1).

**Fig 2:**
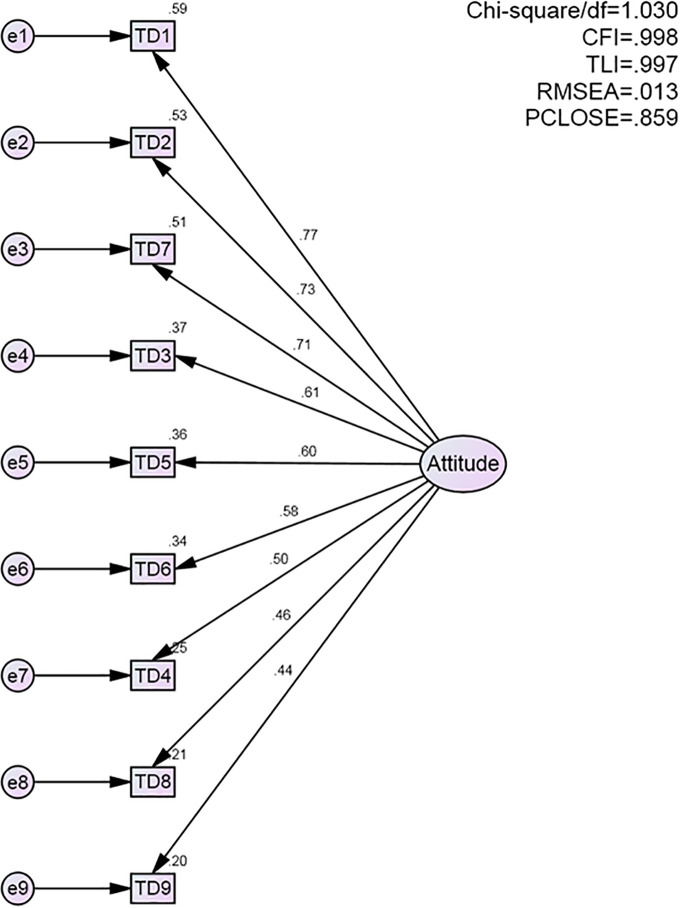
Confirmatory factor analysis model for the attitude domain. Similarly, the practice domain also demonstrated a satisfactory model fit, with CMIN/df = 1.495, CFI = 0.967, RMSEA = 0.054, and PCLOSE = 0.321 (Fig 3).

**Fig 3:**
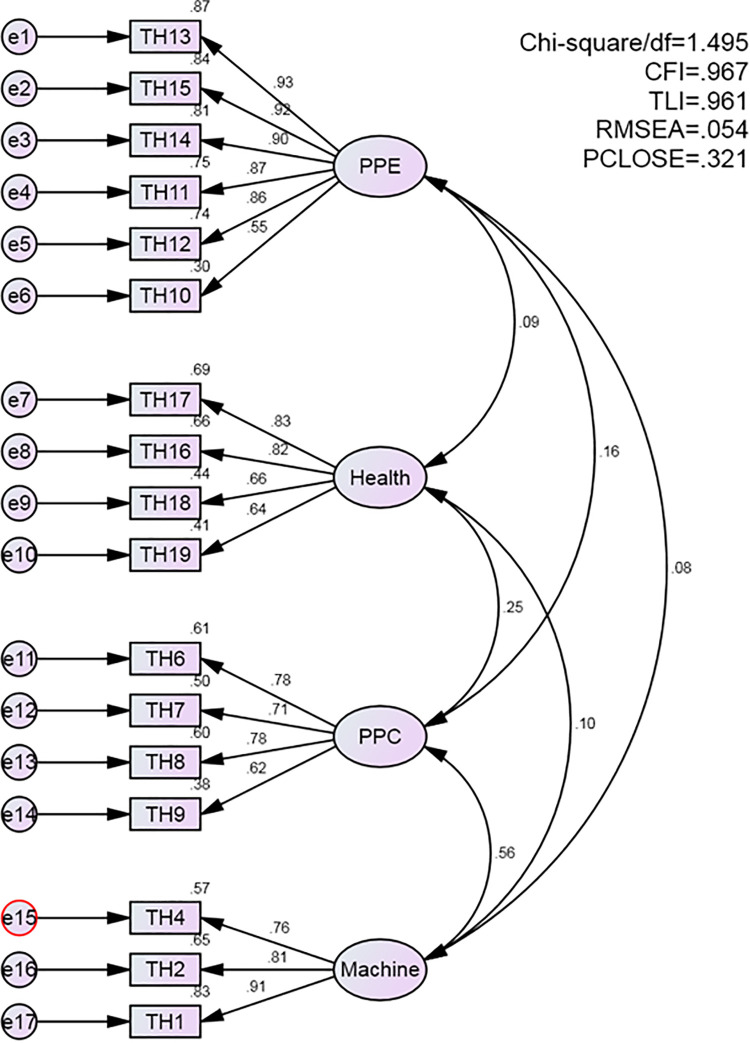
Confirmatory factor analysis model for the practice domain.

## Discussions

The findings of this study demonstrate that rice farmers’ KAP regarding occupational accident prevention are influenced by multiple interrelated factors spanning physical hazards, exposure to agrochemicals, the use of machinery and tools, ergonomic conditions, and animal-related risks. The study aimed to design and validate a comprehensive instrument capable of systematically measuring rice farmers’ KAP related to occupational accident prevention. Both exploratory and confirmatory factor analyses were conducted to assess the validity and reliability of the proposed instrument, which was developed from an initial pool of 50 items.

The results of the EFA and KMO testing confirmed that 46 out of the 50 items were valid indicators for measuring KAP among rice farmers. One item was removed during the face validity stage due to limited cultural relevance, resulting in 49 items entering EFA, all of which were retained after analysis. To the best of our knowledge, this is the first study in Vietnam to develop and validate an instrument specifically designed to assess occupational accident prevention among rice farmers. The instrument underwent a rigorous content validation process involving a multidisciplinary panel of experts in agriculture, trauma care, occupational health, and public health, ensuring both theoretical relevance and contextual appropriateness.

The validated model consists of 46 core indicator variables organized into three latent constructs: knowledge [8,9,19,20,35, 41–43], attitude [18,21,44,45], and practice [24,31,46–50]. The **CFA** indicated that all factor loadings were statistically significant and that the overall model fit indices met acceptable thresholds. These findings affirm that the instrument provides a robust framework for assessing occupational accident prevention behaviors in rice farming and may serve as a reference model for future studies in similar agricultural contexts [20,23,51–53].

Interestingly, the attitude domain yielded a single-factor solution, which, although theoretically coherent, may reflect the homogeneous perception of occupational safety attitudes among rice farmers. This pattern suggests that farmers’ beliefs and motivations toward safety tend to cluster around a unified construct of risk awareness and collective responsibility, rather than multiple nuanced dimensions. However, future studies may consider expanding the number of items or incorporating context-specific attitudinal indicators (e.g., perceived behavioral control or safety self-efficacy) to capture a broader range of psychosocial determinants.

The application of EFA provided valuable insights into the construct validity and dimensional structure of the questionnaire. Adhering to the recommended factor-loading threshold of 0.45 ensured methodological rigor throughout the validation process [54]. The strong KMO values (0.862 for knowledge, 0.890 for attitude, and 0.830 for practice) together with statistically significant Bartlett’s tests (p < 0.001) confirmed the adequacy of the sample for factor analysis. The Cronbach’s alpha coefficients ranged from 0.833 to 0.894, demonstrating high internal consistency across all domains. These reliability levels are comparable to previously validated KAP tools used in agricultural and occupational health research, such as pesticide safety and ergonomic behavior instruments developed in Thailand, India or other countries, which reported α values between 0.80 and 0.90 [20,30–36]. This comparability supports the robustness and transferability of the present instrument across similar rural contexts [55,56]. In addition to internal validity, future studies should evaluate the instrument’s external validity, including test–retest reliability, predictive validity, and responsiveness to behavioral change interventions. Longitudinal testing would further clarify the instrument’s ability to detect temporal changes in farmers’ KAP following training or policy implementation.

The results of the CFA confirmed satisfactory model fit across all domains, with fit indices indicating good structural validity. Specifically, the knowledge domain showed CMIN/df = 1.239, CFI = 0.990, RMSEA = 0.038, and PCLOSE = 0.884; the attitude domain showed CMIN/df = 1.030, CFI = 0.998, RMSEA = 0.013, and PCLOSE = 0.859; and the practice domain demonstrated CMIN/df = 1.495, CFI = 0.967, RMSEA = 0.054, and PCLOSE = 0.321. These values indicate strong construct validity, accuracy, and comprehensiveness of the instrument. The model performance aligns with previous psychometric validation studies emphasizing the value of confirmatory factor analysis in strengthening the theoretical and empirical foundations of measurement tools [56,57].

From a cultural perspective, the interpretation of several items may have been influenced by the social norms and communal work culture typical of rural Vietnamese farmers. For instance, the emphasis on collective safety and mutual assistance may explain the unidimensional structure of the attitude scale. Similarly, local language nuances and idiomatic expressions related to farming practices may shape how respondents perceive occupational risk and personal responsibility. These contextual aspects highlight the importance of cultural adaptation and community engagement when applying standardized measurement tools in low-resource agricultural settings.

Overall, the pilot validation of the developed questionnaire demonstrated strong psychometric properties, including sound content validity, structural validity, and internal consistency reliability. Nevertheless, future research should aim to confirm these findings in larger, more diverse samples and evaluate additional forms of validity (e.g., concurrent and predictive validity). This instrument provides a solid foundation for future large-scale studies assessing farmers’ KAP on occupational accident prevention and offers a standardized tool for evaluating the effectiveness of occupational safety interventions. Beyond measurement utility, the validated KAP framework contributes to promoting a culture of safety and health awareness among rice farmers, thereby supporting broader occupational health initiatives within Vietnam’s agricultural sector. The findings represent a crucial step toward developing evidence-based strategies for improving safety behaviors and reducing occupational risks among rural farming populations.

## Strengths and limitations

The developed instrument possesses several notable strengths that contribute to its validity and reliability. First, the instrument was designed through a rigorous, multi-stage process that combined both quantitative and qualitative approaches. Quantitative analyses, such as exploratory and confirmatory factor analyses and internal consistency testing, were complemented by qualitative evaluations from a multidisciplinary panel of experts in agriculture, occupational health, trauma care, and public health. This integration ensured that the instrument achieved high content validity and accurately reflected the multidimensional nature of knowledge, attitudes, and practices related to occupational accident prevention among rice farmers.

Second, the study adhered to established methodological standards regarding sample size determination, sampling procedures, and expert panel selection, thereby enhancing the scientific rigor and reliability of the results. The structured validation framework, guided by internationally recognized psychometric principles, strengthened the robustness and reproducibility of the instrument.

Despite these strengths, several limitations should be acknowledged. The relatively modest sample size used in this pilot study may constrain the generalizability of the findings to the broader population of rice farmers in Vietnam. In particular, while the sample size of 168 participants was sufficient for EFA, it represents the lower bound of acceptability for CFA, especially given the relatively large number of observed variables. This limitation may affect the stability and precision of some parameter estimates, and future studies with larger samples are recommended to confirm the robustness of the factor structure. Future research employing larger and more diverse samples across different geographic and agricultural contexts would provide stronger evidence for the instrument’s external validity. In addition, while internal consistency and construct validity were thoroughly examined, other psychometric properties, such as test–retest reliability, criterion validity, and predictive validity, were not assessed and should be explored in subsequent research to further substantiate the instrument’s robustness.

## Conclusion

The validated KAP questionnaire provides a reliable and contextually appropriate tool for evaluating occupational safety awareness and behaviors among rice farmers. It holds significant potential for application in future research, surveillance, and intervention programs aimed at improving occupational health and safety in agricultural settings. By providing an evidence-based measurement framework, this study contributes to strengthening preventive strategies, guiding policy development, and fostering a culture of safety within Vietnam’s agricultural workforce.

## Supporting information

S1 File Study data and codebook. (XLSX)
